# Exploring the Impact of Additive Shortcuts in Neural Networks via Information Bottleneck-like Dynamics: From ResNet to Transformer

**DOI:** 10.3390/e26110974

**Published:** 2024-11-14

**Authors:** Zhaoyan Lyu, Miguel R. D. Rodrigues

**Affiliations:** Department of Electronic and Electrical Engineering, University College London, London WC1E 6BT, UK; m.rodrigues@ucl.ac.uk

**Keywords:** deep learning, neural networks, transformer, shortcut connections, information bottleneck theory

## Abstract

Deep learning has made significant strides, driving advances in areas like computer vision, natural language processing, and autonomous systems. In this paper, we further investigate the implications of the role of additive shortcut connections, focusing on models such as ResNet, Vision Transformers (ViTs), and MLP-Mixers, given that they are essential in enabling efficient information flow and mitigating optimization challenges such as vanishing gradients. In particular, capitalizing on our recent information bottleneck approach, we analyze how additive shortcuts influence the fitting and compression phases of training, crucial for generalization. We leverage Z-X and Z-Y measures as practical alternatives to mutual information for observing these dynamics in high-dimensional spaces. Our empirical results demonstrate that models with identity shortcuts (ISs) often skip the initial fitting phase and move directly into the compression phase, while non-identity shortcut (NIS) models follow the conventional two-phase process. Furthermore, we explore how IS models are still able to compress effectively, maintaining their generalization capacity despite bypassing the early fitting stages. These findings offer new insights into the dynamics of shortcut connections in neural networks, contributing to the optimization of modern deep learning architectures.

## 1. Introduction

Machine learning, especially deep learning, has made significant strides in recent years, providing exceptional performance across a variety of tasks and domains [1]. Deep learning has been particularly outstanding in fields like computer vision [2,3,4], natural language processing [5], and autonomous systems [6], where the capacity to model complex patterns from data has revolutionized traditional approaches [1]. These advancements have been driven by increasingly sophisticated neural network architectures, which continue to evolve to address new challenges [7,8].

One of the central innovations within deep learning architectures is the use of different neural network structures designed to optimize performance and efficiency. From the early multi-layer perceptrons (MLPs) [9] to the more advanced convolutional neural networks (CNNs) [3], recurrent neural networks (RNNs) [10], and attention-based architectures like Transformers [5], the evolution of these models has unlocked new capabilities for machine learning systems.

Among these developments, **additive shortcut connections** have emerged as a fundamental architectural feature that enhances the training of deep neural networks. Introduced to mitigate issues like the vanishing gradient problem, shortcut connections enable the flow of information across layers more efficiently [4]. These connections, particularly in architectures like ResNet [4] and its derivatives, allow neural networks to bypass intermediate layers, facilitating the learning process and improving the performance. Shortcut structures are now the go-to choice for most state-of-the-art (SOTA) models, especially in vision-based tasks, and are integral to architectures such as Vision Transformers and MLP-Mixers [11,12].

However, despite the widespread success of these models, our understanding of how these shortcut connections influence the training process remains incomplete. While shortcuts help alleviate optimization challenges, they introduce complexities into how neural networks learn and generalize [13]. Specifically, the way networks with shortcut connections navigate the fitting and compression phases commonly observed in the training process—as described by the information bottleneck theory [14]—remains unclear. This gap in understanding is particularly pronounced for networks like Transformers, where the presence of identity shortcuts allows information to bypass layers, potentially altering the traditional pathways to generalization [5]. The mechanisms through which these models retain and compress information, and the effect of shortcut connections on these processes, have yet to be fully explored.

This paper focuses on exploring the role of additive shortcut connections in modern neural networks, particularly their effect on the training dynamics of models such as ResNet and Transformers. Specifically, we aim to investigate how models equipped with these shortcuts behave during the fitting and compression phases of training, which are critical for the model’s generalization ability. This exploration is grounded in information bottleneck theory [14], which suggests that neural networks aim to increase the information relevant to the ground-truth labels in their representations while reducing irrelevant information [15]. When examining neural network training dynamics through the information bottleneck framework, it has been found that the training process typically involves two key phases: an initial fitting phase, where relevant information is captured, followed by a compression phase, where irrelevant details are discarded [16].

However, the information bottleneck theory and its dynamics are based on mutual information (MI) measures, which are notoriously difficult to estimate for high-dimensional random variables. Our previous work [17] introduced practical measures for analyzing these phases using Z-X and Z-Y metrics, which are based on the minimal mean squared error (MMSE) and conditional entropy, respectively. These metrics allow for a more reliable analysis of the generalization pathways, circumventing the computational challenges of traditional MI-based approaches. In this study, we leverage this framework to examine how these dynamics are affected by the presence of additive shortcut connections, specifically focusing on architectures such as ResNet, Vision Transformers, and MLP-Mixers.

### 1.1. Contributions

This paper makes the following key contributions to the study of neural networks with additive shortcut connections, particularly in the context of understanding their training dynamics:

1. **Identification of distinct fitting and compression behaviors:** We demonstrate that models with *non-identity shortcuts* (such as traditional ResNet architectures) follow the conventional two-phase training process, consisting of an initial fitting phase followed by a subsequent compression phase, similar to the behavior seen in traditional feed-forward networks [17].
  In contrast, models with *identity shortcuts*, such as Vision Transformers and MLP-Mixers, deviate from this pattern by skipping the initial fitting phase and moving directly into the compression phase. This deviation represents a significant challenge to traditional views of neural network optimization, where both fitting and compression are typically expected phases of training.
2. **Analysis of the mechanisms underlying the absence of a fitting phase:** We conjecture that models with identity shortcuts are able to skip the fitting phase because the shortcut structure enables the model to propagate all necessary information for the classification task to deeper layers without the need for early-stage fitting.
  This conjecture is validated empirically by comparing Z-Y measures at initialization. These comparisons show that models with identity shortcuts retain sufficient information for the task even without requiring an explicit fitting phase, indicating that the network can bypass the typical initial training process.
3. **Analysis of the mechanisms driving compression:** We also explore the mechanisms that drive compression in models with identity shortcuts. Through extensive empirical experiments, we identify two distinct mechanisms:
  - In ResNet-like models with identity shortcuts, compression is caused by the *canceling effect* between the so-called functional and informative components (which we will define later) of the network’s representations.
  - In Transformers and MLP-Mixers, compression occurs when the functional component of the network representations *overwhelms* the informative component, leading to a pronounced compression phase.

### 1.2. Organization of the Paper

The paper is organized as follows:

- Section 1 provides an overview of the relevance of deep learning architectures and the importance of additive shortcut connections. It introduces the motivation behind studying the fitting and compression phases in these architectures.
- Section 2 discusses the Transformer architecture, state-of-the-art deep learning models, and shortcut structures in neural networks, with a focus on understanding their generalization and training dynamics.
- Section 3 introduces the Z-X and Z-Y measures adopted in this work, providing a background on their use to observe fitting and compression phases in neural networks.
- Section 4 presents empirical results comparing neural networks with and without shortcut connections, with a particular focus on ResNet, ViT, and MLP-Mixer models. The Z-X dynamics of models with identity and non-identity shortcuts are explored in detail.
- Section 5 analyzes the empirical findings, particularly the reasons why IS models skip the fitting phase and how they manage to compress effectively. This section further explores the interaction between the functional and informative components in network representations.
- Section 6 summarizes the key findings of the paper and outlines potential directions for future research.

### 1.3. Notations

The mathematical notations used in this paper are summarized as follows: We use capital letters such as *X* and *Y* to represent matrices or tensors, where *X* typically denotes input data, and *Y* denotes the corresponding labels or targets. Throughout this paper, we refer to layers in a neural network with subscripted notations, such as $Z_{l}$, where *l* refers to the layer index and *Z* represents the intermediate representation at that layer. The notation $f_{\theta }$ is used to denote functions or transformations parameterized by a set of learnable parameters θ within the neural network.

## 2. Related Work

### 2.1. The Transformer as a State-of-the-Art Deep Learning Architecture

The Transformer model has rapidly become one of the most influential architectures in the field of deep learning, particularly in natural language processing (NLP) [5]. Its success lies in its ability to capture complex dependencies in data using self-attention mechanisms, which allow the model to attend to different parts of an input sequence or image without the limitations of fixed-size receptive fields.

In recent years, the Transformer architecture has been extended beyond NLP into the domain of computer vision, most notably through the development of the **Vision Transformer** (ViT) [11]. The ViT leverages the same self-attention mechanism to process image patches, treating them as sequences, thereby bypassing the need for convolutional layers. ViT has shown state-of-the-art (SOTA) performance on several vision benchmarks, particularly in classification tasks, where it competes with or even surpasses traditional CNN-based architectures like ResNet [4,18].

Despite their widespread adoption, Transformers also present new challenges in terms of training and generalization [19]. Their high computational demand, reliance on large datasets, and potential susceptibility to overfitting raise important questions about their efficiency and scalability [20]. These challenges make it crucial to further investigate the internal mechanisms of Transformers, especially in how they navigate the fitting and compression phases during training. Understanding these dynamics is particularly important as Transformers incorporate identity shortcuts, which may influence the way they handle information compared to more traditional architectures like CNNs.

#### Understanding Transformers

As the Transformer architecture has risen to prominence, a growing body of research has aimed to understand its inner workings, particularly the mechanisms that enable it to generalize so effectively across tasks and domains. These efforts have primarily focused on explaining how the self-attention mechanism and multi-layer structure contribute to the model’s performance and how they differ from more traditional architectures such as CNNs and RNNs [5,21].

One line of research has investigated the self-attention mechanism itself, seeking to explain how it captures long-range dependencies and the role of multi-head attention in learning diverse representations. Studies have shown that attention heads in Transformers often learn different patterns, with some focusing on local relationships while others capture more global dependencies [11,21,22]. This versatility allows Transformers to model complex interactions in the data, but the exact contributions of individual attention heads and layers to the overall learning process remain topics of active investigation.

Another important research direction has focused on the positional encoding component, which provides the necessary ordering information in a model that inherently lacks a sense of sequence [5,23]. Positional encoding is critical for the model’s ability to handle sequential data like language or structured inputs like images (in the case of Vision Transformers). Researchers have explored different types of positional encoding—learned versus fixed—and their impact on the model’s ability to generalize across different types of data [23,24,25,26].

In addition, several studies have attempted to visualize and interpret the internal representations learned by Transformers. Some works have used attention visualization techniques to map out which parts of an input are considered most important by the model [27]. These visualizations help to demystify how Transformers make decisions but also highlight a key challenge: unlike CNNs, where activations can often be linked to specific spatial features, attention maps can be more abstract and harder to interpret in terms of direct feature relationships [28]. This makes understanding the fitting and compression phases of training in Transformers particularly challenging, as their mechanisms for storing and discarding information are less intuitive than those of CNNs.

Finally, the presence of identity shortcuts in Transformers—similar to those found in ResNet—adds another layer of complexity to understanding these models’ generalization dynamics. These shortcuts allow information to flow directly to deeper layers without it undergoing transformation in every block, potentially bypassing important fitting processes that occur in other architectures. The question of how these shortcuts impact the compression of information and what role they play in the model’s overall training dynamics is a central focus of this paper.

### 2.2. Exploring Shortcut Structures in Neural Networks

The introduction of shortcut connections into neural networks was a groundbreaking development that aimed to address the challenges of training deep models, particularly the issue of vanishing gradients. Early neural networks suffered from the inability to propagate gradients effectively across multiple layers, leading to poor optimization, especially in very deep architectures. Shortcut connections, also known as skip connections, were first popularized by the ResNet architecture, which allowed gradients to flow more freely through layers by bypassing intermediate transformations.

In ResNet [4], the key innovation was the use of additive shortcuts, which merge the input of one layer with a deeper layer additively. This architectural modification proved to be crucial in enabling the training of very deep networks with hundreds or even thousands of layers, leading to significantly improved performance on a wide range of tasks. The success of ResNet sparked further exploration into different types of shortcut structures and their implications for network training and performance.

Subsequent research has explored other forms of shortcut connections, such as the concatenation shortcuts used in DenseNet [29]. Unlike the identity shortcuts in ResNet, DenseNet’s concatenation shortcuts combine the outputs of all previous layers, allowing each layer to access not only its immediate predecessors but also distant layers’ outputs. This creates a more densely connected architecture that encourages feature reuse and leads to more compact models, as fewer parameters are needed to achieve the same performance levels. These concatenation shortcuts have been shown to enhance the learning efficiency of networks, particularly in scenarios with limited data.

Beyond ResNet and DenseNet, modern architectures like Vision Transformers (ViTs) and MLP-Mixers have also incorporated shortcut connections, though in different ways [11,12,30]. In these models, shortcut connections play a critical role in maintaining stability and enabling the effective training of very deep networks. The additive shortcuts in Vision Transformers, for instance, allow information to be passed across layers without any transformation, which we later referred to as identity shortcuts. 

While shortcut connections have undeniably eased the training process and improved the performance of deep networks, their role in the fitting–compression dynamic remains an area of active research. Understanding how these connections impact the flow of information through networks, and how they affect the model’s ability to fit and compress data during training, is crucial to optimizing these architectures for different tasks.

### 2.3. Methodologies for Understanding Neural Networks

In the effort to demystify the behavior of neural networks, several methodologies have been developed and applied to gain insights into their operations. These methods can be categorized into three primary approaches: explainable deep learning, generalization bound analysis, and network dynamics analysis.

#### 2.3.1. Explainable Deep Learning

Explainable deep learning focuses on making neural network decisions more transparent by visualizing the internal representations or learned features at different layers of the model. Early studies in this area predominantly centered on feature visualization, where methods like saliency maps and activation mapping were used to highlight which parts of an input, such as an image, had the greatest influence on the network’s prediction [31]. Techniques such as Grad-CAM and its variants have been widely adopted for this purpose, offering a glimpse into which regions of an input are emphasized during inference [27,32,33,34,35].

However, these visual explanations are often limited to specific input examples and do not provide a complete understanding of the network’s behavior across the entire dataset or in different operational conditions. As a result, while explainability techniques contribute valuable insights into how networks handle individual samples, they are insufficient for analyzing the broader generalization capabilities of the model. This has led to the pursuit of more comprehensive methods for understanding neural networks from a probabilistic or statistical perspective.

#### 2.3.2. Generalization Bound Analysis

Generalization bounds provide a more theoretical approach, using statistical learning theory to describe how well a model trained on a given dataset is likely to perform on unseen data. This method relies on concepts such as the Vapnik–Chervonenkis (VC) dimension and PAC learning to derive the bounds on a model’s performance [36,37,38,39]. These approaches help quantify the ability of a model to generalize by estimating how much information a model retains from the training data and how that affects its performance on new data.

While generalization theory has yielded important insights, there are several limitations to its practical application. For instance, deriving tight bounds for deep neural networks, especially large models with many layers, remains a challenge [40]. Additionally, while generalization bounds can describe the potential behavior of a model, they do not fully capture the dynamic process of training, particularly how models transition from fitting the data to effectively generalizing across unseen instances.

#### 2.3.3. Information Bottlenecks and Network Dynamics Analysis

The information bottleneck (IB) theory has emerged as a powerful framework for understanding the learning dynamics of neural networks, providing insights into how models generalize by balancing the retention of relevant information (fitting) and the discarding of irrelevant details (compression) [14,15]. Initially developed in the context of information theory, the IB principle posits that neural network learning can be conceptualized as a process of capturing and retaining only essential information for the task at hand while compressing redundant or irrelevant features [14]. This two-phase dynamic, marked by an initial fitting phase followed by a compression phase, is crucial to understanding how neural networks transition from learning to generalizing on unseen data [15].

A growing body of research has used the IB framework to analyze how information flows through layers during training. A notable study by Tishby et al. [16] introduced mutual information as a tool for tracking the information flow within neural networks, illustrating that they generally exhibit an early period of fitting, where useful information is retained, followed by an extended phase of compression, where unnecessary details are discarded. This framework has provided theoretical support for why deeper networks often generalize better despite their higher capacity to overfit, suggesting that effective compression enhances their generalization capabilities. However, estimating mutual information in high-dimensional spaces, as required for complex networks with many parameters and layers, poses significant challenges [41,42,43]. These challenges have led to varying findings in the literature, with some studies observing strong compression phases and others reporting minimal or even absent compression [13,16,44,45,46,47].

To address these limitations, recent work has proposed more computationally feasible alternatives to mutual information, such as MMSE-based Z-X measures and conditional-entropy-based Z-Y measures [17]. These metrics offer a practical way to observe the network dynamics without the complexities associated with mutual information estimation. Studies leveraging Z-X and Z-Y measures have reaffirmed the presence of fitting and compression phases in simpler architectures like feed-forward neural networks while also extending this analysis to complex structures such as CNNs and MLPs.

Building on these foundations, this paper explores how additive shortcut connections in architectures like ResNet and Transformers influence the generalization dynamics. While the IB theory has helped reveal how deep networks generally manage information retention and compression, the impact of shortcut connections on these processes is less understood. By extending IB analysis to models with additive shortcuts, this work aims to clarify how these connections affect the training dynamics, particularly in modern architectures such as Vision Transformers and MLP-Mixers. Additionally, the use of Z-X and Z-Y measures offers a practical framework for overcoming some of the computational challenges associated with traditional IB approaches, facilitating a more detailed examination of how shortcut connections navigate the fitting and compression phases to improve generalization [17].

## 3. Recap: The Z-X Measure and the Z-Y Measure for Observing the Fitting and Compression Phases

In this paper, we adopt the Z-X and Z-Y measures proposed in our previous work [17] to study the dynamics of neural networks with additive shortcuts. As defined in [17], we refer to the networks under investigation (such as CNNs, Transformers, and MLP-Mixers) as the **subject networks**. This section briefly introduces the definitions of Z-X and Z-Y measures and their associated dynamics.

We focus on image classification problems, using *X* to represent the image input and *Y* as the ground-truth labels. The intermediate representation of layer *l* is denoted as $Z_{l}$. Formally, we model the classification with a Markov chain as follows:

$$\begin{array}{c} Y\to X\to Z_{1}\to \cdots \to Z_{l}\to \cdots \to Z_{L} \end{array}$$

The representation at the last layer $Z_{L}$ corresponds to the network’s prediction.

In this Markov chain structure, *Y* is positioned at the beginning because it represents the target label information that flows through the network. This setup reflects the progression of information from the ground-truth label *Y* to the input *X* and subsequently through each layer’s representation $Z_{l}$. Placing *Y* at the start of the chain is essential in the IB theory and related analyses, as it ensures that $Z_{L}$ (and all intermediate representations $Z_{l}$) encodes information about *Y* derived through *X*. This Markov chain structure captures the dependency relationships needed for IB analysis, where the information learned by $Z_{l}$ is traceable to *Y* via *X*, reinforcing that each layer’s representation gradually refines the information needed for classification.

### 3.1. The Z-X Measure and the Z-Y Measure

In our previous work, we introduced two key measures—Z-X and Z-Y measures—to provide more reliable insights into neural network dynamics, specifically focusing on the **fitting** and **compression phases**. These measures serve as alternatives to mutual information (MI), which is difficult to estimate in high-dimensional settings. The Z-X and Z-Y measures are computationally feasible.

#### 3.1.1. The Z-X Measure

The Z-X measure is the MMSE (minimum mean squared error) between the neural network representation *Z* and the input data *X*, estimated by a neural-network-based estimator in a data-driven manner. More specifically, given a dataset $D=\left(X_{1},Y_{1}\right),∆,\left(X_{\left|D\right|},Y_{\left|D\right|}\right)$, we estimate the Z-X measure $m_{Z;X}$ as follows:

(1) $$\begin{array}{cc} m_{Z;X} & =\mathrm{mmse}(Z|X)\\ \; & \approx \min_{\phi }\frac{1}{\left|D\right|}\sum_{k=1}^{\left|D\right|}{\left(f_{\phi }\left(Z_{l;k}\right)-X_{k}\right)}^{⊤}\left(f_{\phi }\left(Z_{l;k}\right)-X_{k}\right)\\ \; & =\frac{1}{\left|D\right|}\sum_{k=1}^{\left|D\right|}{\left(f_{\phi^{*}}\left(Z_{l;k}\right)-X_{k}\right)}^{⊤}\left(f_{\phi^{*}}\left(Z_{l;k}\right)-X_{k}\right)\\ \; & =\frac{1}{\left|D\right|}\sum_{k=1}^{\left|D\right|}{\left(f_{\phi^{*}}\circ f_{\theta_{1:l}}\left(X_{k}\right)-X_{k}\right)}^{⊤}\left(f_{\phi^{*}}\circ f_{\theta_{1:l}}\left(X_{k}\right)-X_{k}\right)\;, \end{array}$$

where $f_{\theta_{1}}\left(X_{k}\right)$ represents the subject network’s forward pass from layer 1 to layer *l* for the input sample $X_{k}$, and $Z_{l;k}$ is the corresponding representation generated by the subject network at layer *l* for the input sample $X_{k}$. $f_{\phi }$ is a separate estimator network, parameterized by ϕ, trained to reconstruct the input $X_{k}$ from the intermediate representation of the subject network $Z_{l;k}$. The Z-X measure quantifies how much of the original input information is retained by the network at layer *l*.

#### 3.1.2. The Z-Y Measure

The Z-Y measure captures how well the neural network representation $Z_{l}$ at layer *l* can predict the target label *Y*. It is defined as the conditional entropy H(Y|Z), which quantifies the uncertainty in predicting label *Y* given representation $Z_{l}$. The Z-Y measure is estimated similarly to the Z-X measure, using a neural-network-based predictor $f_{\psi }\left(Z_{l;k}\right)$ for the label $Y_{k}$. Specifically, it is defined as follows:

(2) $$\begin{array}{cc} m_{Z;Y} & =H(Y|Z)\\ \; & \approx \min_{\psi }\frac{1}{\left|D\right|}\sum_{k=1}^{\left|D\right|}\ell_{\mathrm{CE}}\left(f_{\psi }\left(Z_{l;k}\right),Y_{k}\right)\\ \; & =\frac{1}{\left|D\right|}\sum_{k=1}^{\left|D\right|}\ell_{\mathrm{CE}}\left(f_{\psi^{*}}\left(Z_{l;k}\right),Y_{k}\right)\\ \; & =\frac{1}{\left|D\right|}\sum_{k=1}^{\left|D\right|}\ell_{\mathrm{CE}}\left(f_{\psi^{*}}\circ f_{\theta_{1:l}}\left(X_{k}\right),Y_{k}\right), \end{array}$$

where $f_{\psi }$ is a separate estimator network parameterized by ψ, trained to predict the label $Y_{k}$ from the internal representation $Z_{l;k}$. $\ell_{\mathrm{CE}}$ represents the cross-entropy loss function.

### 3.2. Estimating the Z-X and Z-Y Measures

**Figure 1:**
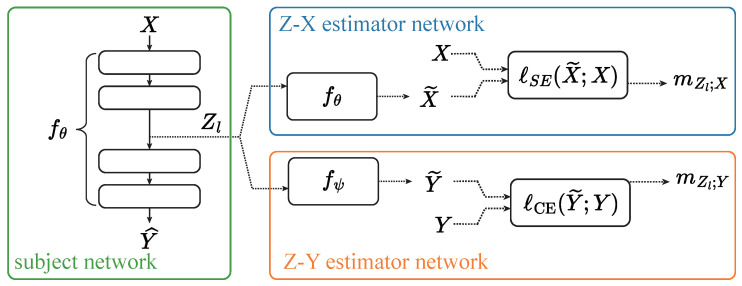
The framework for estimating the Z-X and Z-Y measures. This figure is adapted from Figure 1 in [17]. $\ell_{SE}$ refers to the squared loss, and $\ell_{CE}$ represents the cross-entropy loss.

The estimation of the Z-X and Z-Y measures involves training separate neural networks to minimize the squared loss (for the Z-X measure) and the cross-entropy loss (for the Z-Y measure). These processes are shown in Figure 1 and can be described as follows:

**Z-X estimation:** For each input $X_{k}$, the subject network’s forward pass generates the representation $Z_{l;k}$ at layer *l*. This representation is passed through the Z-X estimator network $f_{\phi }$, which is trained to reconstruct $X_{k}$ by minimizing the mean squared error (MSE) between $f_{\phi }\left(Z_{l;k}\right)$ and the original input $X_{k}$, as shown in Equation (1).

**Z-Y estimation:** Similarly, to estimate the Z-Y measure, the representation $Z_{l;k}$ is passed through the Z-Y estimator network $f_{\psi }$, which is trained to predict the target label $Y_{k}$ by minimizing the cross-entropy loss, as shown in Equation (2).

To address potential overfitting, we split the dataset D used for Z-X and Z-Y estimation into two subsets: $D_{\mathrm{train}}$ and $D_{\mathrm{valid}}$ (Unless otherwise specified, we split the dataset D into $D_{\mathrm{train}}$ and $D_{\mathrm{valid}}$ with a 7:3 ratio in this study). The training subset $D_{\mathrm{train}}$ is used to train the estimator networks, while the validation subset $D_{\mathrm{valid}}$ ensures that the estimators do not overfit. The final Z-X and Z-Y measures are computed based on the validation set, ensuring robust estimation. Both estimators are trained using a gradient-descent-based algorithms until convergence, identified when the estimated measures on the validation set cease to decrease. At this point, the final Z-X and Z-Y measures are obtained based on the validation set for each layer *l* of the subject network. The complete training procedure for estimating the Z-X and Z-Y measures is outlined in Algorithm 1.    
**Algorithm 1:** Estimate the Z-X measure and the Z-Y measure with the validation set.
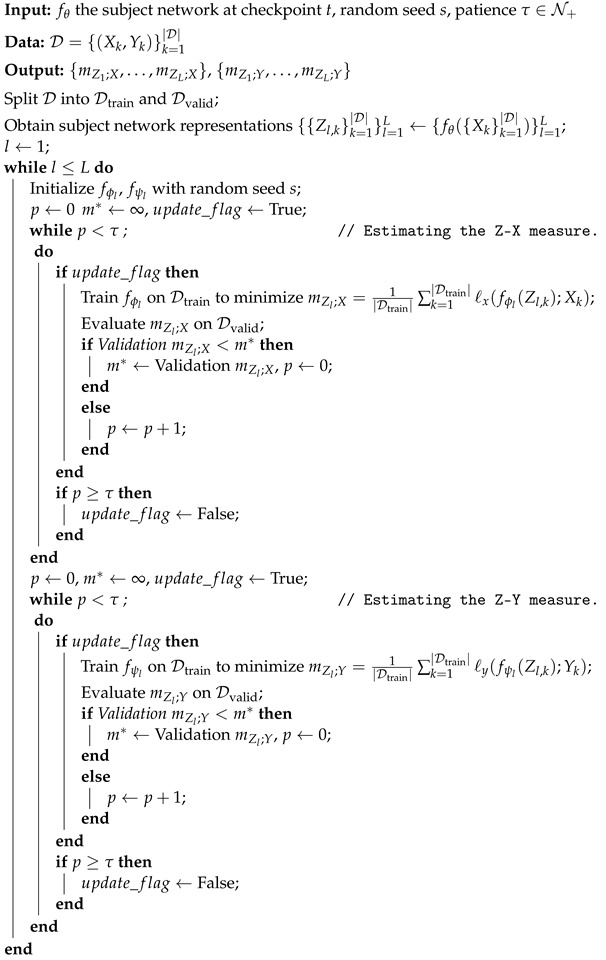


Specific details regarding the estimator network architecture and training setups will be provided in the relevant sections.

### 3.3. Z-X Dynamics and Z-Y Dynamics

By plotting the Z-X and Z-Y measures for each layer across different stages of training, we can gain valuable insights into how the subject network’s internal representations evolve during training. In doing so, at various checkpoints during the training of the subject network, we freeze the parameters of the subject network. We estimate the Z-X and/or Z-Y measures for the current state of the network. Each checkpoint provides a point in the Z-X/Z-Y space, representing the network’s ability to retain input information (with the Z-X measure) and predict labels (with the Z-Y measure) during training.

Tracking the Z-X and Z-Y measures over multiple checkpoints allows us to plot the **Z-X dynamics** and **Z-Y dynamics** of the subject network. This will reveal how the network transitions between the fitting and compression phases.

### 3.4. The Fitting Phase and the Compression Phase

As highlighted in our previous work [17], neural networks generally undergo two distinct phases during training: the **fitting phase** and the **compression phase**.

#### 3.4.1. The Fitting Phase

In the early part of training, the network typically undergoes a fitting phase, where it captures as much information about the input data as possible. During this phase, the Z-X measure decreases as the network’s intermediate layers are learning to retain more information about the input *X*, while the Z-Y measure decreases as the network learns to predict the labels effectively.

#### 3.4.2. The Compression Phase

After the fitting phase, the network enters the compression phase, where it discards unnecessary information. This phase is marked by an increase in the Z-X measure, indicating reduced retention of input information, while the Z-Y measure stabilizes as the network retains the most relevant features for accurate label prediction.

The presence of these two phases during neural network training, as theorized by the information bottleneck theory, has been a topic of much discussion and debate [13,16,44,45,46,47]. In this work, we extend the exploration of these phases, focusing specifically on neural networks with additive shortcuts.

## 4. The Fitting and Compression Phases in Models with Additive Shortcuts

### 4.1. Identity Shortcuts and Non-Identity Shortcuts

**Figure 2:**
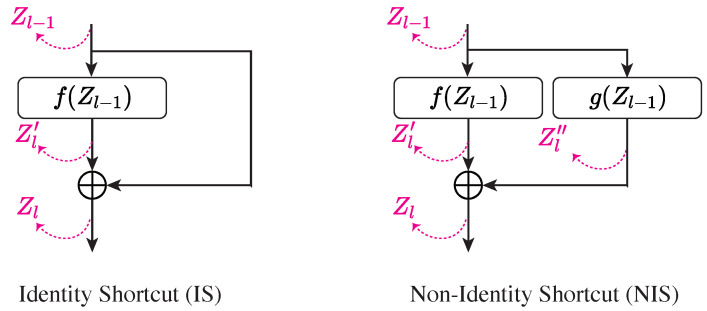
An illustration of identity and non-identity shortcuts. The representations at different stages are labeled in pink.

We categorize additive shortcuts into two types: identity shortcuts (ISs) and non-identity shortcuts (NISs). Figure 2 illustrates the structure of both types.

**Identity shortcuts (ISs)** refer to connections where the input is passed directly to a deeper layer without any transformation. These shortcuts “skip” layers, allowing the input to flow through the network unmodified, preserving information across multiple layers. ISs can only be used in models where the input and output dimensions of a layer are the same, enabling seamless addition to the output. ISs are fundamental in architectures such as Transformers and MLP-Mixers. Mathematically, an IS can be expressed as

$$\begin{array}{c} Z_{l}=f\left(Z_{l-1}\right)+Z_{l-1}=Z_{l}^{\prime }+Z_{l-1}, \end{array}$$

where $f(Z_{l-1})$ represents the transformation in the main path.

**Non-identity shortcuts (NISs)**, in contrast, apply transformations to the input before passing it to deeper layers. Such transformations, like convolutions or linear operations, are often necessary when the input and output are in different dimensions, as seen in models like ResNet [4]. For instance, in ResNet, NISs are used when downsampling layers reduce the spatial dimensions. In these cases, a convolutional transformation ensures the shortcut can be added to the main path’s output without dimension mismatches.

Mathematically, a NIS can be written as

$$\begin{array}{c} Z_{l}=f\left(Z_{l-1}\right)+g\left(Z_{l-1}\right)=Z_{l}^{\prime }+Z_{l}^{\prime \prime }. \end{array}$$

where $g(Z_{l-1})$ is the transformation applied to the input from layer l−1, and $f(Z_{l-1})$ is the main path’s transformation.

In this section, we examine and compare the Z-X dynamics of five neural networks:

- **(NS) CNN:** A VGG-like CNN serves as the control group with no shortcuts (NS).
- **(NIS) ResCNN:** A ResCNN model incorporating NISs, similar to ResNet [4].
- **(IS) iResCNN:** A modified ResCNN, incorporating ISs throughout.
- **(IS) Vision Transformer (ViT):** A ViT model that uses ISs by design.
- **(IS) MLP-Mixer:** A MLP-Mixer model also with ISs by design.

### 4.2. The CNN and ResCNN

#### 4.2.1. Setup

**Figure 3:**
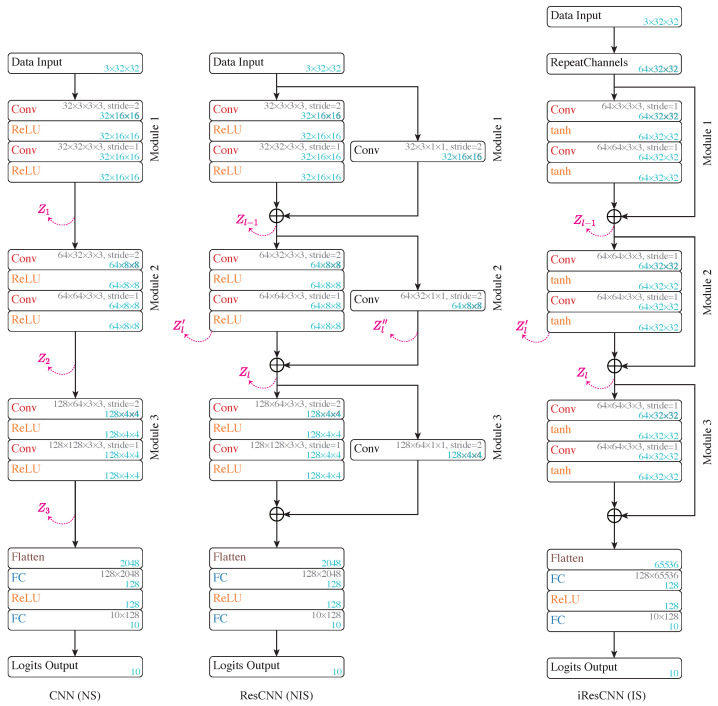
Architecture of the CNN (**left**), ResCNN (**middle**), and iResCNN (**right**). “Conv” refers to convolutional layers, “ReLU” to rectified linear unit activation, and “FC” to fully connected layers. The convolutional kernel and weight matrix shapes are noted in gray, and the tensor/matrix/vector shapes are labeled in blue.

**Subject network setup**: We begin by analyzing the Z-X dynamics of a CNN and a residual CNN (ResCNN). Figure 3 (left and middle) illustrates the architectures. This experiment aims to compare the dynamics of networks without shortcuts (NS) and with NISs, laying the foundation for analyzing models with ISs.

As shown in Figure 3, both models feature three modules, each doubling the depth while halving the representation shape. The ResCNN’s main path shares the CNN’s architecture, with each module adopting a NIS. The shortcut in the ResCNN uses a convolutional layer (with a kernel size of 1) to align the input’s shape, allowing for additive merging.

We investigate the Z-X dynamics of $Z_{1}$, $Z_{2}$, and $Z_{3}$ in the CNN and the corresponding $Z_{l}$ layers in the ResCNN.

Both models are trained on the CIFAR-10 dataset [48] (The information bottleneck theory has traditionally faced difficulties in estimating mutual information for high-dimensional representations, leading many studies to rely on synthetic data [16] or simpler datasets such as MNIST [49]. In contrast, the MMSE-based Z-X measure and conditional-entropy-based Z-Y measure adopted in this paper are more computationally feasible, allowing us to work with higher-dimensional data.) for 50 epochs, with a batch size of 256. The training uses cross-entropy loss with the AdamW optimizer, with a weight decay of 1.0 and a learning rate of 0.0001. No data augmentation is applied. This configuration ensures an efficient training process without pronounced overfitting.

**Figure 4:**
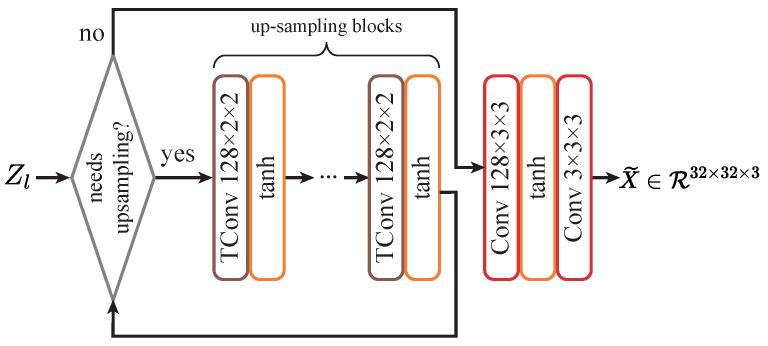
Z-X estimator design for convolutional networks. “TConv” stands for transposed convolution, used to upscale feature maps, and “tanh” represents the hyperbolic tangent activation function.

**Z-X estimator setup**: To estimate Z-X measures, the estimator must map the representation back to the input image space. We use the design shown in Figure 4. This setup ensures stable and swift estimation of the Z-X measures.

To track the Z-X dynamics, we freeze the subject network’s parameters at various checkpoints, feed the full CIFAR-10 training set through the network to obtain representations, and then optimize the Z-X estimator. The Z-X estimator is trained using the Adam optimizer with a learning rate of 0.0001 for up to 200 epochs, with early stopping after 10 epochs of no test loss improvement.

#### 4.2.2. The Z-X Dynamics of the CNN and ResCNN

**Figure 5:**
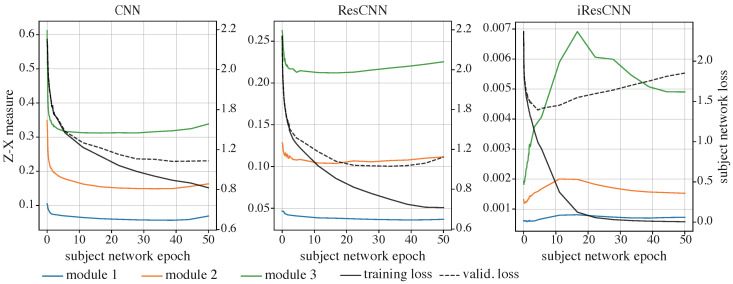
The Z-X dynamics of the CNN (**left**), ResCNN (**middle**), and iResCNN (**right**). The Z-X measures are estimated at corresponding modules in Figure 3.

The Z-X dynamics of the CNN and ResCNN are shown in Figure 5 (left and middle). Both networks exhibit a fitting phase (where Z-X measures decrease), followed by a compression phase (where the Z-X measures increase), consistent with previous observations in feed-forward neural networks [17].

### 4.3. The iResCNN

#### 4.3.1. Setup

**Subject network setup**: The architecture of the iResCNN is detailed as follows: Firstly, the number of channels is increased from 3 to 64 by cyclically repeating the red, green, and blue channels. This approach ensures that there is no information loss when increasing the dimensional representation at the beginning of the neural network. Subsequently, within each residual module, the number of channels and the feature map size remain unchanged, allowing for the implementation of identity shortcuts, as the feature maps consistently maintain a 64×32×32 dimension. Each residual block includes two convolutional layers, each succeeded by a tanh activation function. The feature maps are eventually flattened, followed by fully connected layers functioning as the classification head, similar to the ResCNN. The precise architecture of the iResCNN is depicted in the right panel in Figure 3.

The iResCNN is trained and validated on the standard CIFAR-10 dataset, with all the training hyper-parameters mirroring those used for the CNN and ResCNN.

**Z-X estimator setup**: The architecture of the estimator network for the Z-X dynamics and the training setups for the estimator adhere to the configuration established for the ResCNN.

#### 4.3.2. The Z-X Dynamics of the iResCNN

The right panel in Figure 5 displays the Z-X dynamics of $Z_{l}$ from various residual modules in the specially designed iResCNN.

Notably, this configuration of the residual network largely skips the initial fitting phase, launching directly with a compression phase. In comparison with the control group—the Z-X dynamics of the CNN and ResCNN presented in the left and middle panels in Figure 5—it is safe to conjecture that this behavior is primarily due to the inclusion of ISs within the network.

Following the initial compression, a decline in the Z-X measure in $Z_{l}$ of modules 2 and 3 is observed. This subsequent fitting phase, however, coincides with the network exhibiting mild signs of overfitting, as indicated by an increase in the validation set loss.

Overall, during the decrease in the subject network’s validation loss, replacing the NISs in the ResCNN with ISs appears to result in Z-X dynamics that bypass the initial fitting phase and commence with a compression phase.

### 4.4. ViTs and MLP-Mixers

ViTs and MLP-Mixers diverge significantly in their structure from CNNs and MLPs. Both of them initially tokenize the input data and then process them through a series of modules featuring ISs. In particular, the ViTs are composed of a sequence of MHSA and FF modules, while MLP-Mixers are composed of a sequence of token-mixers and channel-mixers. The representation maintains a consistent shape throughout the ViTs and MLP-Mixers, up to the classification head, comprising a tensor with *n* tokens, each of *d* dimensions. Hence, the additive shortcuts applied to the MHSA, FF, token-mixer and channel-mixer modules in ViTs and MLP-Mixers qualify as ISs.

#### 4.4.1. Setup

**Figure 6:**
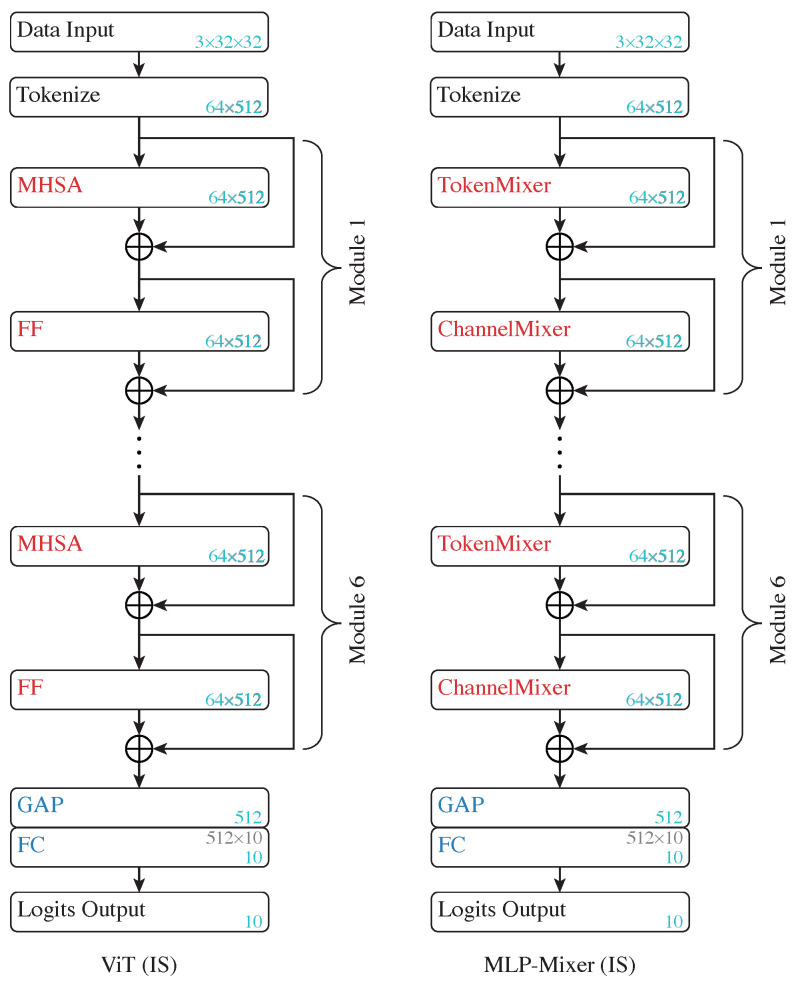
Architecture of ViT (**left**) and MLP-Mixer (**right**). “MHSA” represents multi-head self attention modules, “FF” indicates feed-forward modules, and “GAP” represents global average pooling layers.

**Subject network setup**: The ViT implemented is constituted of six Transformer modules, and the architecture is shown in the left panel in Figure 6.

The model first segments the image input into patches (64 patches per image, 4×4 pixels per patch), which are subsequently embedded into tokens (each with a length of 512) through a learnable embedding module. Additive sine–cosine positional encoding is then utilized. Each MHSA module accommodates eight heads and maintains a dropout rate of 0.1. Note that the ViT does not use a class token. Instead, it adopts global average pooling (GAP) to reduce the token dimensions before feeding it to a linear classification head, as implemented in [18].

The MLP-Mixer—as shown in the right panel in Figure 6—employs the same tokenization as the ViT model and contains six token-mixer and channel-mixer modules, with the token dimensions also fixed at 512. The token-mixer and channel-mixer setups are aligned with the original implementation in [12].

Both the ViT and the MLP-Mixer are trained from scratch on the standard CIFAR-10 dataset using the same recipe: Using the Adam optimizer, the learning rate is initially set to 0.001 and is gradually reduced to 0 following a cosine scheduling pattern [50]. The training period spans 500 epochs, and the batch size is configured to 2048. To augment the training data, an initial application of random augmentation [51] is employed, with the number of operations set to 2 and the magnitude at 14. This is followed by padding the image with 4 pixels on each side and randomly cropping it back to the original size (32×32). A random horizontal flip is also performed. For regularization, we implement Mixup regularization [51] with a hyper-parameter of 0.2, CutMix regularization [52] is also set at a hyper-parameter of 0.2, and label smoothing [53] is used with a hyper-parameter of 0.3. The weight decay parameter is set to 0.001.

This training setup enables the ViT and MLP-Mixer models to be trained from scratch on the CIFAR-10 dataset, achieving performance levels that approach the state-of-the-art benchmarks [54]. Specifically, our ViT model reached an accuracy of 86.14%, while the MLP-Mixer achieved 83.76%, demonstrating effective training and meaningful representation learning.

**Z-X estimator setup**: Compared with the subject networks previously examined that use convolutional layers as the main building blocks, the ViT and MLP-Mixer architectures are more memory-intensive in their implementation [11]. The tokenized representations in the ViT, with a dimensionality of 64 × 512 = 32,768, require full matrix multiplications for each pairwise token interaction. This contrasts with convolutional networks, which are more memory-efficient due to their ability to use shared kernels across spatial locations, reducing the memory intensity and allowing for dimensionality reduction through operations like pooling.

As a result, it is crucial to design the estimators for measuring the Z-X dynamics of ViTs and MLP-Mixers to be lightweight. Otherwise, it would be challenging to efficiently implement both the ViT and MLP-Mixer subject networks and the associated estimators on a server to estimate the Z-X measures effectively.

**Figure 7:**
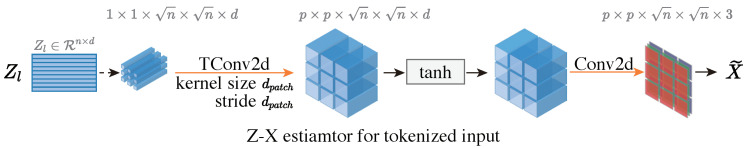
Architecture of Z-X estimators for token-based models. For the tokenized representation of the ViT and MLP-Mixer in this paper, n=64, p=8, and d=512.

As depicted in Figure 7, the Z-X estimator for the ViT and MLP-Mixer begins with reordering of the tokens. Since each token in the ViT and MLP-Mixer representation is initially flattened and embedded from an image patch, this approach intuitively aligns each token with its corresponding image patch. In particular, the tokenize module in the ViT and MLP-Mixer subject networks divides the square CIFAR-10 input image into 64 patches, each embedded into vectors with a length of 512. Consequently, the reordered representation has a shape of 1×1×8×8×512.

This step is followed by the application of a transposed convolution operation [55], designed to increase the height and width of the representation to match those of the input data. More specifically, for the ViT and MLP-Mixer models trained on the CIFAR-10 dataset, the initial patch size is 4×4. Therefore, the output of the transposed convolution operation is shaped as 4×4×8×8×512.

Then, a tanh activation function is applied, followed by a convolutional layer aiming to adjust the channel of the representation to ensure the output of the estimator matches the original input data. This design considers the shape of the representation, making the estimator network lightweight yet stable for optimization.

#### 4.4.2. The Z-X Dynamics of the ViT and MLP-Mixer

**Figure 8:**
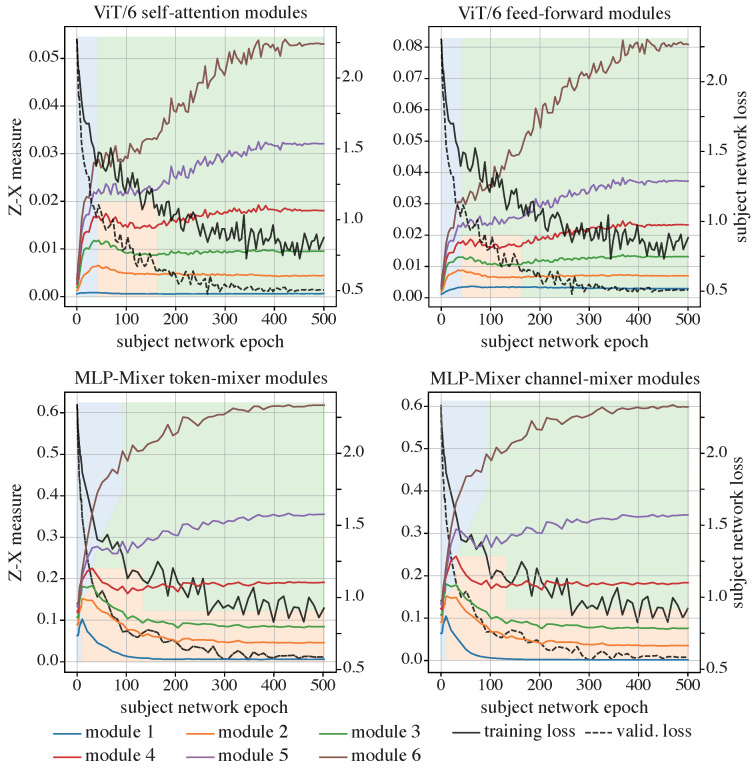
The Z-X dynamics of the ViT (**top**) and MLP-Mixer (**bottom**). The Z-X measures are estimated at the corresponding modules in Figure 6.

It can be observed from the upper panels in Figure 8 that the ViT, when trained from scratch, also skips the initial fitting phase, commencing instead with an immediate compression phase. Following this, some of the layers experience a mild fitting phase, subsequently leading into another phase of compression observable across all layers, characterized by a slow increase in the Z-X measure. Notably, the Z-X measure corresponding to the output of the first Transformer module remains near zero throughout the duration of training. This stability can be attributed to its direct connection to the input embeddings via an IS.

When compared with the iResCNN (the right panel in Figure 5), which is also trained on the CIFAR-10 dataset, the Z-X dynamics of the ViT begin from a comparable value at epoch 0 but exhibit a much more aggressive increase than those of the iResCNN.

In the lower panels of Figure 8, the findings within the MLP-Mixer parallel those noted in the ViT, where the network demonstrates an initial compression phase rather than a fitting phase. Following this initial compression, the early layers transition into a fitting phase, whereas the deeper layers persist in their compression efforts. Both channel-mixers and token-mixers follow similar trends.

Overall, models with ISs generally skip the initial fitting phase and instead exhibit a pronounced compression phase, distinguishing them from neural networks that do not implement shortcuts or that use NISs. Meanwhile, we observe that while ViT models display a milder fitting phase, MLP-Mixer models exhibit a more pronounced fitting phase in their shallower layers. Although the cause of this difference is not entirely clear, we have documented it here for transparency. Despite these variations, all models with ISs retain the ability to compress, suggesting that compression remains essential for generalization in neural networks with ISs.

## 5. Why Does the Behavior of IS-Based Models Differ from the Behavior of NIS-Based Models?

The investigation of the Z-X dynamics in models with additive shortcuts—particularly those with identity shortcuts (ISs)—has highlighted two distinct phenomena: (1) models with ISs tend to skip the initial fitting phase during training, and (2) models with ISs still exhibit pronounced compression phases.

This section further explores these findings by addressing two key questions: (1) why do models with ISs skip the initial fitting phase, and (2) how are models with ISs able to compress effectively despite skipping the fitting phase?

### 5.1. On the Absence of the Initial Fitting Phase

We conjecture that the absence of the initial fitting phase is due to the compression of the nature of the IS: at random initialization, the model featuring an IS is able to pass almost all the information necessary for the classification task to the deeper layers. This means the network does not need an explicit fitting phase to help the representation in the deep layers to gain information about the ground-truth labels and can start the compression phase directly to achieve generalization.

We designed an experiment to empirically validate our conjecture. The information captured by the representations about the ground-truth labels can be quantified using the Z-Y measure, which is estimated by minimizing Equation (2). If the Z-Y measure is sufficiently low at the random initialization stage (i.e., before any training), we can safely claim that our conjecture is validated.

But what constitutes a “sufficiently” low Z-Y measure? Fortunately, our Z-Y measure is based on minimizing the cross-entropy loss between the estimator’s output and the ground-truth labels. This is directly comparable to the loss function used by the subject network in the classification task—the subject networks are trained to minimize the cross-entropy loss between their predictions and the ground-truth labels. Hence, we use the lowest cross-entropy loss of the subject network, denoted as $\ell_{\theta^{*}}\left(X;Y\right)$, as the threshold for judging the Z-Y measure. If the Z-Y measure, $m_{Z_{l};Y}$, is comparable to or lower than $\ell_{\theta^{*}}\left(X;Y\right)$, we assert that the representation has gained sufficient information about the ground-truth labels.

#### 5.1.1. Estimating Z-Y Measures

We estimate the Z-Y measures for the models at random initialization (i.e., checkpoint 0), with a particular focus on the representations from the deeper layers (closer to the output). According to the data processing inequality, if the deeper layers possess sufficient information about the ground-truth labels, the earlier layers should as well.

The Z-Y estimators for the CNN, ResCNN, and iResCNN share similar architectures. They use the ResCNN architecture shown in the middle panel in Figure 3, with the first convolutional layer adapting to the representation’s channel dimension. For the ViT and MLP-Mixer, we employ the ViT model (without the tokenization layer) directly as the Z-Y estimator.

Regarding the optimization setup, the Z-Y estimators for the CNN, ResCNN, and iResCNN mirror the training setups (in terms of the choice of optimizer, learning rate, regularization, and data augmentation) used for the ResCNN subject network. Similarly, the Z-Y estimators for the ViT and MLP-Mixer follow the same setup as those used for training the ViT subject network.

#### 5.1.2. Results

Table 1 presents the Z-Y measures for various subject networks at random initialization.

The results support our conjecture. As shown in Table 1, the models without ISs (i.e., the CNN and ResCNN) show Z-Y measures higher than $\ell_{\theta^{*}}\left(X;Y\right)$. In contrast, the models with ISs (the iResCNN, ViT, and MLP-Mixer) exhibit Z-Y measures lower than $\ell_{\theta^{*}}\left(X;Y\right)$. This empirical validation confirms our conjecture that ISs can propagate sufficient information about the ground-truth labels at random initialization.

### 5.2. The Mechanisms Enabling Compression in Models with ISs

As previously outlined, the models equipped with ISs throughout are able to transmit information from the input data to deeper layers easily. However, pronounced compression behaviors are evident in the representations generated by these models. Therefore, this section delves into the composition of these representations, identifying and discussing various scenarios that might lead to compression behavior.

To begin, we propose decomposing the representations into two components—which are the *functional component* and the *informative component*—in order to understand their interaction. We then propose several element-wise statistics to investigate the interplay between the two components and to try to understand the mechanisms behind the compression phenomena of the models featuring ISs.

#### 5.2.1. Methodology

**Decomposition of the representation of a sub-network with ISs**: Let us formulate a module with ISs as follows:

$$\begin{array}{cc} Z_{l} & =f_{\theta_{l}}\left(Z_{l-1}\right) \end{array}$$

(3) $$\begin{array}{cc} \; & =h_{\theta_{l}}\left(Z_{l-1}\right)+Z_{l-1} \end{array}$$

(4) $$\begin{array}{cc} \; & =Z_{l}^{\prime }+Z_{l-1}. \end{array}$$

Consider a sub-network represented as $f_{\theta_{1:l}}=f_{\theta_{l}}\circ \cdots \circ f_{\theta_{1}}$, where each module features identity shortcuts, as formulated in Equations (3) and (4). The representation $Z_{l}$ can be decomposed in the following manner:

$$\begin{array}{cc} Z_{l} & =f_{\theta_{1:l}}\left(I\left(X\right)\right)\\ \; & =f_{\theta_{l}}\circ f_{\theta_{l-1}}\circ \cdots \circ f_{\theta_{1}}\left(I\left(X\right)\right)\\ \; & =h_{\theta_{l}}\circ f_{\theta_{l-1}}\circ \cdots \circ f_{\theta_{1}}\left(I\left(X\right)\right)+f_{\theta_{l-1}}\circ \cdots \circ f_{\theta_{1}}\left(I\left(X\right)\right)\\ \; & =h_{\theta_{l}}\circ f_{\theta_{l-1}}\circ \cdots \circ f_{\theta_{1}}\left(I\left(X\right)\right)+h_{\theta_{l-1}}\circ f_{\theta_{l-2}}\circ \cdots \circ f_{\theta_{1}}\left(I\left(X\right)\right)+\cdots +h_{\theta_{1}}\left(I(X)\right)+I\left(X\right) \end{array}$$

(5) $$\begin{array}{cc} \; & =\sum_{i=1}^{l}h_{\theta_{i}}\left(Z_{i-1}\right)+I\left(X\right) \end{array}$$

(6) $$\begin{array}{cc} \; & =\sum_{i=1}^{l}Z_{i}^{\prime }+Z_{I} \end{array}$$

(7) $$\begin{array}{cc} \; & =Z_{l;F}+Z_{I}, \end{array}$$

where $Z_{0}=Z_{I}$. I(X) is a pre-processing function that applies to the input *X* and does not reduce any information in *X*. For example, the tokenization layers in the Transformers can be viewed as one such function.

In Equation (7), the representation $Z_{l}$ is effectively decomposed into two components: $Z_{l;F}$, the *functional component*, which is the sum of all $Z_{l}^{\prime }$ processed by the respective residual branches in the residual modules, and $Z_{I}$, the *informative component*, which is a propagation of the pre-processed input I(X).

We now define several element-wise measures to help us investigate the interactions between the functional component and the informative component.

**Element-wise mean and variance**: Consider three random tensors X,Y, and $Z\in R^{h\times w\times c}$, with Z=X+Y. We use $X_{i,j,k}$, $Y_{i,j,k}$, and $Z_{i,j,k}$ to denote specific elements in the three-dimensional tensors *X*, *Y* and *Z*, respectively.

Each element in a tensor, such as $X_{i,j,k}$, can be treated as a scalar random variable. The variance in this scalar, denoted as $\sigma_{X_{i,j,k}}^{2}$, is defined as the element-wise variance, reflecting the variability in $X_{i,j,k}$ across different instances. Similarly, the element-wise mean, $\mu_{X_{i,j,k}}$, represents the average value of $X_{i,j,k}$. Formally, the element-wise mean and variance are defined as follows:

$$\begin{array}{cc} \mu_{X_{i,j,k}} & =E[X_{i,j,k}]\\ \sigma_{X_{i,j,k}}^{2} & =E[{\left(X_{i,j,k}-\mu_{X_{i,j,k}}\right)}^{2}]. \end{array}$$

**Element-wise covariance and the correlation coefficient**: To assess the relationship between corresponding elements in tensors *X* and *Y*, the element-wise covariance between corresponding elements in *X* and *Y* is calculated as follows:

$$\begin{array}{c} \mathrm{Cov}\left(X_{i,j,k},Y_{i,j,k}\right)=E\left[\left(X_{i,j,k}-\mu_{X_{i,j,k}}\right)\left(Y_{i,j,k}-\mu_{Y_{i,j,k}}\right)\right]. \end{array}$$

In turn, the (Pearson’s) correlation coefficient between the corresponding elements in *X* and *Y* is given by

$$\begin{array}{c} \mathrm{Corr}\left(X_{i,j,k},Y_{i,j,k}\right)=\frac{\mathrm{Cov}(X_{i,j,k},Y_{i,j,k})}{\sigma_{X_{i,j,k}}\sigma_{Y_{i,j,k}}}, \end{array}$$

where $\sigma_{X_{i,j,k}}$ and $\sigma_{Y_{i,j,k}}$ are the standard deviations of $X_{i,j,k}$ and $Y_{i,j,k}$, respectively. This correlation is a standardized measure of a linear relationship that ranges between −1 and 1.

Given that each $Z_{i,j,k}$ is the sum of $X_{i,j,k}$ and $Y_{i,j,k}$, the element-wise variance in $Z_{i,j,l}$ can be expressed as follows:

$$\begin{array}{c} \sigma_{Z_{i,j,k}}^{2}=\sigma_{X_{i,j,k}}^{2}+\sigma_{Y_{i,j,k}}^{2}+2\mathrm{Cov}\left(X_{i,j,k},Y_{i,j,k}\right). \end{array}$$

We can also synthesize these element-wise variances in the tensors into overall measures to capture general trends in these statistics across a representation tensor. This can be achieved by computing the averaged element-wise variance and covariance across all elements in tensor *Z* as follows:

(8) $$\begin{array}{cc} \bar{\sigma_{Z}^{2}} & =\frac{1}{h\cdot w\cdot c}\sum_{i=1,j=1,k=1}^{h,w,c}\left(\sigma_{X_{i,j,k}}^{2}+\sigma_{Y_{i,j,k}}^{2}+2\cdot \mathrm{Cov}\left(X_{i,j,k},Y_{i,j,k}\right)\right)\\ \; & =\frac{1}{h\cdot w\cdot c}\left(\sum_{i=1,j=1,k=1}^{h,w,c}\sigma_{X_{i,j,k}}^{2}+\sum_{i=1,j=1,k=1}^{h,w,c}\sigma_{Y_{i,j,k}}^{2}+2\sum_{i=1,j=1,k=1}^{h,w,c}\mathrm{Cov}\left(X_{i,j,k},Y_{i,j,k}\right)\right)\\ \; & =\bar{\sigma_{X}^{2}}+\bar{\sigma_{Y}^{2}}+2\bar{\mathrm{Cov}(X,Y)}, \end{array}$$

where $\bar{\sigma_{X}^{2}}$ and $\bar{\sigma_{Y}^{2}}$ are the averaged element-wise variances in all individual elements in tensors *X* and *Y*, respectively, and $\bar{\mathrm{Cov}(X,Y)}$ is the averaged element-wise covariance of the corresponding elements across tensor *X* and *Y*.

Likewise, the averaged element-wise correlation coefficient is defined as follows:

$$\begin{array}{c} \bar{\mathrm{Corr}(X,Y)}=\frac{1}{h\cdot w\cdot c}\sum_{i=1,j=1,k=1}^{h,w,c}\bar{\mathrm{Corr}(X_{i,j,k},Y_{i,j,k})}. \end{array}$$

Substituting *X* with $Z_{l,F}$, the functional component, and *Y* with $Z_{I}$, the informative component, gives us $Z=Z_{l}$, the representation of the sub-network $h_{\theta_{1:l}}$ with identity shortcuts.

**Dynamics of the element-wise statistics**: Given the same dataset, the representations generated by a neural network may change as the parameters are updated through the learning algorithm. We aim to study how the statistics—the averaged element-wise variance and the averaged element-wise correlation coefficient—evolve as the network undergoes training. The dynamics of the element-wise statistics will be analyzed as a function of the training checkpoints.

More specifically, having decomposed the output of the sub-network with identity shortcuts $Z_{l}$ into a functional component $Z_{l;F}$ and an informative component $Z_{I}$, we introduce the notations $Z_{l}^{\left(t\right)}$, $Z_{l;F}^{\left(t\right)}$, and $Z_{I}^{\left(t\right)}$ to represent the values obtained from the neural network at checkpoint *t*. The subsequent sections will analyze how the aforementioned element-wise statistics evolve as a function of *t*.

#### 5.2.2. Results and Analysis

**Figure 9:**
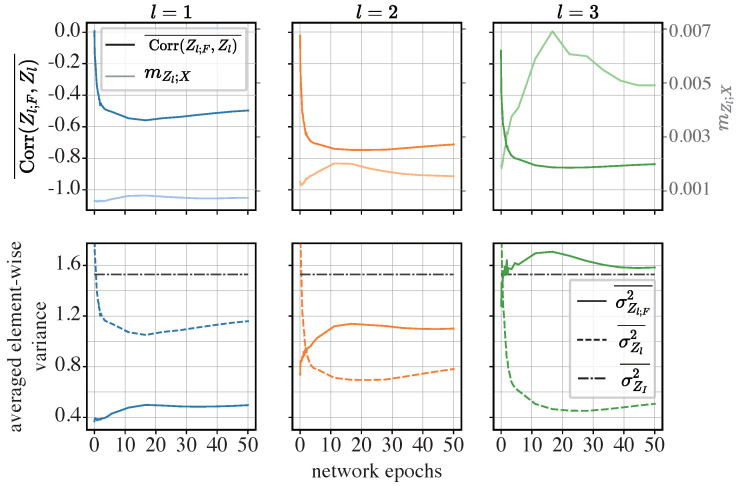
Dynamics of averaged element-wise correlation coefficients and averaged element-wise variance in the iResCNN: In the upper row, the curves in a darker color and the left axis show the dynamics of $\bar{\mathrm{Corr}(Z_{l;F},Z_{I})}$, while the lighter curves and the right axis show the Z-X measure ($m_{Z_{l};X}$) obtained from Section 4.2. In the lower row, the dynamics of $\bar{\sigma_{Z_{l}}^{2}}$, $\bar{\sigma_{Z_{l;F}}^{2}}$, and $\bar{\sigma_{Z_{I}}^{2}}$ are visualized. The left, middle, and right panels show the representations of different modules. *l* is the index for the modules in the iResCNN shown in the right panel of Figure 3. Panels in the same row or column share the same axes.

**The iResCNN**: First, we analyze the dynamics of the element-wise correlation coefficient and the element-wise variance in the iResCNN. The results are displayed in Figure 9.

The upper row in Figure 9 shows that $\bar{\mathrm{Corr}(Z_{l;F},Z_{I})}$ is closely correlated with the Z-X dynamics (plotted with $m_{Z_{l};X}$), which reflect the magnitude of compression. More specifically, from epoch 0 to epoch 15, $\bar{\mathrm{Corr}(Z_{l;F},Z_{l})}$ decreases as $m_{Z_{l};X}$ increases. However, from the 15th epoch onwards, the compression tends to be less pronounced as $m_{Z_{l};X}$ decreases mildly, while $\bar{\mathrm{Corr}(Z_{l;F},Z_{I})}$ also increases after the 15th epoch. Additionally, the deeper module (e.g., l=3) exhibits lower $\bar{\mathrm{Corr}(Z_{l;F},Z_{I})}$, corresponding to higher $m_{Z_{l};X}$.

From the lower row in Figure 9, we observe that the averaged element-wise variance in the functional component $\bar{\sigma_{Z_{l;F}}^{2}}$ is higher in deeper layers and tends to increase as the network exhibits compression (referencing the upper row in Figure 9). Notably, in the last module (l=3), $\bar{\sigma_{Z_{l;F}}^{2}}$ exceeded $\bar{\sigma_{Z_{I}}^{2}}$. This suggests that the functional components $Z_{l;F}$ play an increasingly important role in the overall representation $Z_{l}$ as the network deepens and exhibits compression.

Additionally, we find that the trends in $\bar{\sigma_{Z_{l;F}}^{2}}$ seem to be negatively correlated with $\bar{\sigma_{Z_{l}}^{2}}$, consistent with the relationship shown in Equation (8) when substituting X,Y, and *Z* with $Z_{I},Z_{I;F},$ and $Z_{l}$, respectively. More specifically, Equation (8) states

$$\begin{array}{c} \bar{\sigma_{Z_{l}}^{2}}=\bar{\sigma_{Z_{I}}^{2}}+\bar{\sigma_{Z_{l;F}}^{2}}+2\bar{\mathrm{Cov}(Z_{I},Z_{l;F})}. \end{array}$$

Here, $\bar{\sigma_{Z_{I}}^{2}}$ is constant. If $\bar{\sigma_{Z_{l}}^{2}}$ decreases while $\bar{\sigma_{Z_{l;F}}^{2}}$ increases, this implies a decrease in $\bar{\mathrm{Cov}(Z_{I},Z_{l;F})}$. In fact, $\bar{\mathrm{Cov}(Z_{I},Z_{l;F})}$ has become negative, as indicated by the negative $\mathrm{Corr}(Z_{l};Z_{l;F})$, which essentially is a normalized measure of the covariance.

Finally, $\bar{\sigma_{Z_{l;F}}^{2}}$ and $\bar{\sigma_{Z_{l}}^{2}}$ are roughly on the same scale as $\bar{\sigma_{Z_{I}}^{2}}$, indicating that the representation is not dominated by either the functional or informative components. This contrasts with the trends observed in the ViT and MLP-Mixer, which will be discussed later.

These patterns suggest that the pronounced compression in the iResCNN may be due to the network learning to make the functional components more negatively correlated with the informative components, implying a potential canceling effect.

**Figure 10:**
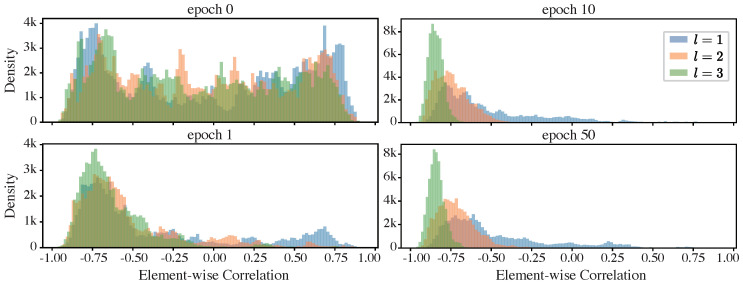
Histograms of element-wise correlation coefficients in the iResCNN: These histograms summarize the element-wise coefficients $\mathrm{Corr}(Z_{l;F;i},Z_{I;i})$, where *i* indexes the entries of the representation components.

To further validate this conjecture, we examined histograms of the element-wise correlation coefficient between the functional and informative components across different modules and network epochs, as shown in Figure 10.

The histograms corroborate our conjectures. At initialization (epoch 0), the elements in the functional and informative components can be either positively or negatively correlated, with the element-wise correlation coefficients spread from −1 to 1. As training progresses (from epoch 1 to epoch 10), an increasing number of elements show negative correlations. By epoch 10, when the network exhibits considerable compression (refer to the upper row in Figure 9, to the curves in a lighter color with the right axis), a significant portion of the elements displays a correlation close to −1. At the end of training, the level of compression is less pronounced compared to that at the 10th epoch, with the correlation coefficients for most elements showing a slight deviation away from −1.

Comparing different layers, the elements in the representations of the deeper layers show stronger negative correlations, indicating a more significant canceling effect.

Our observations support the hypothesis that canceling effects in the iResCNN, which we identified as a potential source of compression in the representations generated by sub-networks with identity shortcuts, play a crucial role. Conventionally, identity shortcuts are used to facilitate the training of very deep neural networks. However, our results suggest that these networks may inherently discover a mechanism for compression through cancellation. This finding is significant, as compression is crucial for enhancing generalization in neural networks.

**The MLP-Mixer**: We conducted the same experiments on the MLP-Mixer. In particular, we chose to present the last three residual modules, as they exhibit the most pronounced compression.

In the fourth module of the MLP-Mixer (l=4), the elements in the functional components tend to be negatively correlated with the informative component, and as the representation exhibits more pronounced compression, the correlation decreases. This pattern is consistent with what was observed in the iResCNN, implying a canceling effect.

However, in the fifth (l=5) and sixth (l=6) modules, only in the first five epochs do the averaged correlation coefficients decrease as compression increases. In later epochs, while the Z-X measure continues to increase, the correlation coefficient stops decreasing and stabilizes at a certain negative value.

**Figure 11:**
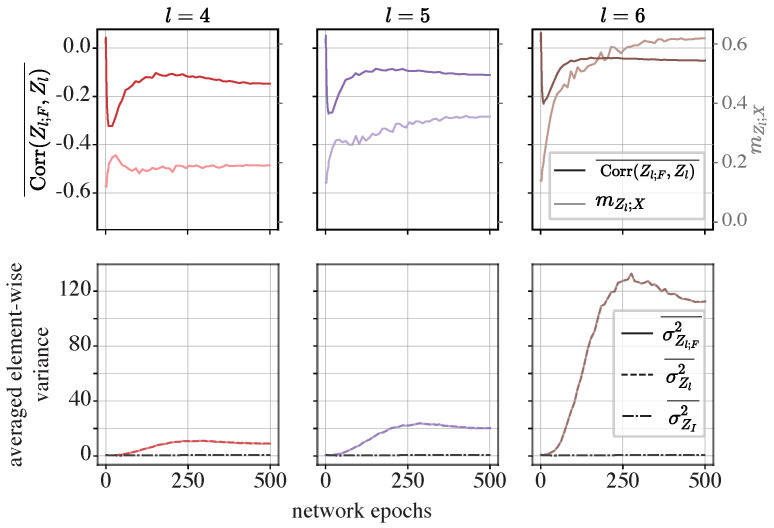
Dynamics of the averaged element-wise correlation coefficients and averaged element-wise variance in the MLP-Mixer: In the upper row, the curves in a darker color and the left axis show the dynamics of $\bar{\mathrm{Corr}(Z_{l;F},Z_{I})}$, while the lighter curves and the right axis show the Z-X measure ($m_{Z_{l};X}$) obtained from Section 4.4. In the lower row, the dynamics of $\bar{\sigma_{Z_{l}}^{2}}$, $\bar{\sigma_{Z_{l;F}}^{2}}$, and $\bar{\sigma_{Z_{I}}^{2}}$ are visualized. The left, middle, and right panels show the representations of different modules. *l* is the index for the modules in the MLP-Mixer shown in the right panel in Figure 6. Panels in the same row or column share the same axes.

Referring to the lower row in Figure 11, it is evident that the averaged element-wise variance in the summed output $Z_{l}$ is dominated by the functional component $Z_{l;F}$, as $\bar{\sigma_{Z_{l}}^{2}}$ closely aligns with $\bar{\sigma_{Z_{l;F}}^{2}}$, with both being significantly higher in scale than $\bar{\sigma_{Z_{I}}^{2}}$. This suggests that the informative components may be overwhelmed by the functional components, potentially complicating the reconstruction of the input data from the summed output. Using a neural-network-based estimator to estimate the MMSE, the dominance of the functional components may bias the estimation of the true MMSE, resulting in a positive Z-X measure. Nevertheless, this observation provides insight into why reconstructing the input data from the representation becomes increasingly difficult: the variance in the representation is dominated by the functional components, which are processed by neural network layers.

In contrast, this dominance of $Z_{l;F}$ in $\sigma_{Z_{l}}^{2}$ was not observed in the iResCNN. More specifically, the lower row in Figure 9 shows that $\sigma_{Z_{l;F}}^{2}$ and $\sigma_{Z_{l}}^{2}$ are on the same scale as $\sigma_{Z_{I}}^{2}$, and the variance in $\sigma_{Z_{l;F}}^{2}$ decreases compared to that of $\sigma_{Z_{I}}^{2}$, rather than them evolving in synchronization.

**Figure 12:**
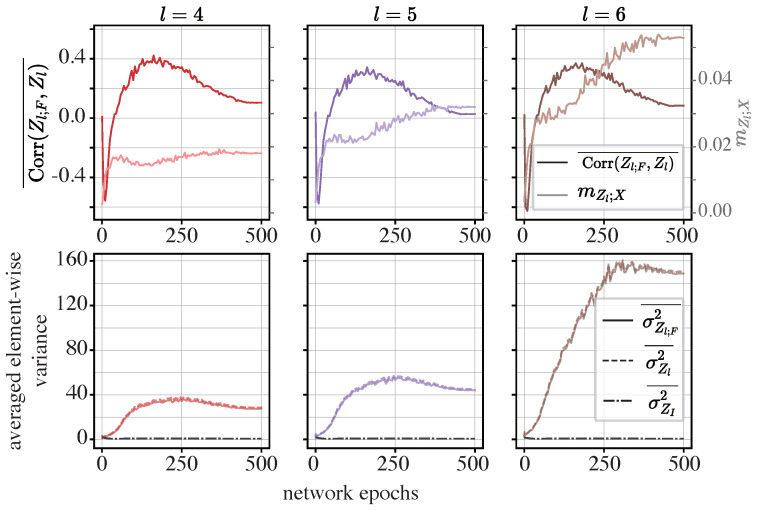
Dynamics of averaged statistics for the ViT: In the upper row, the curves in a darker color and the left axis show the dynamics of $\bar{\mathrm{Corr}(Z_{l;F},Z_{I})}$, while the lighter curves and the right axis show the Z-X measure ($m_{Z_{l};X}$) obtained from Section 4.4. In the lower row, the dynamics of $\bar{\sigma_{Z_{l}}^{2}}$, $\bar{\sigma_{Z_{l;F}}^{2}}$, and $\bar{\sigma_{Z_{I}}^{2}}$ are visualized. The left, middle, and right panels show the representations of different modules. *l* is the index for the modules in the ViT shown in the left panel in Figure 6. Panels in the same row or column share the same axes.

**The ViT**: Finally, we examine the ViT model, focusing on the dynamics of averaged the element-wise covariance, Z-X measure, and averaged element-wise variance, as illustrated in Figure 12. Particular attention is given to the last three MHSA modules in the ViT, which exhibit the most pronounced compression phases.

We can observe from Figure 12 that the fourth, fifth, and sixth MHSA modules display trends similar to those in the sixth module of the token mixer in the MLP-Mixer. Specifically, $\bar{\mathrm{Corr}(Z_{l;F},Z_{I})}$ decreases during the initial five epochs, implying a canceling effect, and then increases rapidly and eventually becomes positive during subsequent epochs.

Meanwhile, $\bar{\sigma_{Z_{l}}^{2}}$ and $\bar{\sigma_{Z_{l;F}}^{2}}$ increase significantly from the fifth epoch onwards, surpassing $\bar{\sigma_{Z_{I}}^{2}}$ in magnitude. The trends in $\bar{\sigma_{Z_{l}}^{2}}$ and $\bar{\sigma_{Z_{l;F}}^{2}}$ are also well synchronized.

These observations suggest that the initial compression phase is driven by a canceling effect. However, as the network is trained over more epochs, the element-wise variances in the functional components overwhelm those of the informative components, indicating a shift in the network’s internal dynamics.

Both the MLP-Mixer and the ViT make a point similar to that for the iResCNN and conventional feed-forward neural networks investigated in [17], where the networks discover a compression mechanism that may be useful for generalization (despite the fact that they use shortcuts to improve training). This, in effect, means that shortcuts do not impair generalization.

## 6. Conclusions

This paper focuses on neural networks with additive shortcuts, examining their fitting and compression phenomena in learning dynamics. Empirical experiments show that models with non-identity shortcuts (NISs), such as the ResCNN, exhibit an initial fitting phase followed by a subsequent compression phase, similar to neural networks without additive shortcuts examined in the prior literature. However, in neural networks with identity shortcuts (ISs), such as the iResCNN, ViT, and MLP-Mixer, the initial fitting phase is notably absent, and these models instead display pronounced compression phases.

Further analysis in this study investigates the mechanisms underlying the observed behaviors in models with ISs. We conjecture and empirically validate that the absence of the initial fitting phase may be due to the ability of ISs to forward sufficient information about the ground-truth labels even at random initialization of the subject network.

Additionally, the pronounced compression phase in models with ISs appears to arise from different underlying mechanisms. For the iResCNN, the compression throughout training primarily results from a cancellation effect within its functional components. In ViTs and MLP-Mixers, the situation is more complex. Initially, the functional components also exhibit cancellation effects on the informative components; however, as the training progresses, the functional components increasingly dominate, overwhelming the informative components and resulting in significant data compression. These insights are crucial, as they deepen our understanding of the compression mechanisms in networks with identity shortcuts and suggest potential strategies, such as compression-oriented regularization, to enhance the network efficiency and performance.

### 6.1. Limitations and Future Work

While this study provides valuable insights into information bottleneck dynamics in networks with additive shortcuts, it has certain limitations that should be addressed in future work:

- **The dataset and task scope**: This study uses a single dataset (CIFAR-10) and does not examine other data types or tasks, such as language or video processing, which may exhibit different IB dynamics. Testing these findings across larger and more diverse datasets, especially those with varied data types, is essential to determine the generality of these dynamics.
- **Inductive bias in the ViT and MLP-Mixer**: As noted in the comments, the ViT and MLP-Mixer models are known to have weaker inductive biases than convolutional models, making them less optimal for small datasets like CIFAR-10 when they are trained from scratch. While we trained the ViT and MLP-Mixer to competitive levels of 86.14% and 83.76% accuracy, respectively, these results are still lower than the benchmarks achieved by convolutional models like ResNet. This limitation highlights the need for testing on larger datasets or using pre-trained versions of these models to capture the shortcut path dynamics better.
- **Shortcut configurations**: This study focuses on additive shortcuts and does not explore alternative configurations, such as the concatenation shortcuts used in DenseNet or combinations of identity and non-identity shortcuts. Investigating these different shortcut designs may reveal additional dynamics that influence IB behavior in novel ways.
- **Layer-specific analysis**: The compression behavior in IS-based models appears to vary across layers, suggesting that layer-wise analysis could yield more precise insights into IB dynamics. Future studies could benefit from examining shortcut path dynamics at the layer-specific level to identify potential opportunities to fine-tune the shortcut configurations and improve performance.
- **Memory constraints and computational intensity**: Due to the memory-intensive nature of the ViT and MLP-Mixer, our study required substantial memory resources to implement multiple estimations across layers, checkpoints, and measures (Z-X and Z-Y). Convolutional networks are comparatively more memory-efficient due to shared kernel use and pooling operations, which reduce the memory intensity. In contrast, ViTs’ tokenized representation and dense matrix multiplications increase the computational demands, making these models more challenging to study. Future work could explore memory-optimized methods for self-attention or alternative ways to handle large-scale estimations.

### 6.2. Summary

In summary, this paper extends the landscape of information bottleneck dynamics by exploring models with additive shortcuts and their unique learning phenomena. Despite using a single dataset, the patterns observed align with IB theory and the related literature, suggesting that they may generalize to broader contexts. Future work could build on this foundation by addressing the limitations noted here, testing these dynamics across more datasets, configurations, and applications to deepen our understanding of neural networks with additive shortcuts.

## Figures and Tables

**Table 1:** Z-Y measures compared with the best cross-entropy loss in the subject network. $Z_{-1}$ and $Z_{-2}$ refer to the representations from the last module and the second to last module, respectively. Differences between the Z-Y measure and the lowest subject network loss are shown in red (higher) and green (lower).

	CNN		ResCNN		iResCNN		ViT		MLP-Mixer	
$\ell_{\theta^{*}}\left(X;Y\right)$	1.082		1.049		1.393		0.479		0.571	
$m_{Z_{-2};Y}$	1.354	+0.272	1.252	+0.203	1.376	−0.017	0.326	−0.153	0.446	−0.125
$m_{Z_{-1};Y}$	1.470	+0.388	1.313	+0.264	1.380	−0.013	0.328	−0.151	0.448	−0.123

## Data Availability

The data presented in this study are available on request from the corresponding author.
